# Influences of degree inhomogeneity on average path length and random walks in disassortative scale-free networks

**DOI:** 10.1063/1.3094757

**Published:** 2009-03-30

**Authors:** Zhongzhi Zhang, Yichao Zhang, Shuigeng Zhou, Ming Yin, Jihong Guan

**Affiliations:** 1 School of Computer Science, Fudan University, Shanghai 200433, China; 2 Shanghai Key Laboratory of Intelligent Information Processing, Fudan University, Shanghai 200433, China; 3 Department of Computer Science and Technology, Tongji University, 4800 Cao’an Road, Shanghai 201804, China

## Abstract

Various real-life networks exhibit degree correlations and heterogeneous structure, with the latter being characterized by power-law degree distribution $P\left(k\right)∼k^{-\gamma }$, where the degree exponent γ describes the extent of heterogeneity. In this paper, we study analytically the average path length (APL) of and random walks (RWs) on a family of deterministic networks, recursive scale-free trees (RSFTs), with negative degree correlations and various γ∊(2,1+ln 3/ln 2], with an aim to explore the impacts of structure heterogeneity on the APL and RWs. We show that the degree exponent γ has no effect on the APL d of RSFTs: In the full range of γ, d behaves as a logarithmic scaling with the number of network nodes N (i.e., d∼ln N), which is in sharp contrast to the well-known double logarithmic scaling (d∼ln ln N) previously obtained for uncorrelated scale-free networks with 2≤γ<3. In addition, we present that some scaling efficiency exponents of random walks are reliant on the degree exponent γ.

## I. INTRODUCTION

The last decade has witnessed tremendous activities devoted to the characterization and understanding of real-life systems in nature and society.^1–4^ Extensive empirical studies have revealed that most real networked systems exhibit scale-free behavior,^5^ which means that these systems follow a power-law degree distribution $P\left(k\right)∼k^{-\gamma }$ with degree exponent γ∊ [2,3] Generally, we call a network with scale-free behavior a *scale-free network* (SFN), which has a heterogeneous structure encoded in the characteristic degree exponent γ: the smaller the γ, the stronger the heterogeneity of the network structure. The inhomogeneous degree distribution of a SFN has a profound effect on almost all other aspect of the network structure. For example, it has been established that scale-free behavior is relevant to average path length^6^ (APL) in uncorrelated random SFNs, i.e., the APL d(N) for a network with node number N depends on γ:^7,8^ when γ=3, d(N)∼ln N; when 2≤γ<3, d(N)∼ln ln N.

As known to us all, the ultimate goal of studying network structure (e.g., degree distribution) is to understand how the dynamical behaviors are influenced by the underlying topological properties of the networks.^3,4^ Among many dynamical processes, a random walk on networks is fundamental to many branches of science and engineering and has been the focus of considerable attention.^9–15^ As a fundamental dynamical process, the random walk is related to various other dynamics such as transport in media,^16^ disease spreading,^17^ target search,^18^ and so on. On the other hand, the random walk is useful for the study of network structure, in particular, for the APL.^9,15^ It is thus of theoretical and practical interests to study a random walk on complex networks, revealing how the structure (e.g., structural heterogeneity) affects the diffusive behavior of the random walk.

In addition to the scale-free behavior, it has also been observed that real networks display ubiquitous degree correlations among nodes,^19–21^ which are usually measured by two quantities, i.e., average degree of nearest neighbors of nodes with a given degree^20^ and Pearson correlation coefficient,^21^ both of which are equivalent to each other. Degree correlations are important in characterizing network topology, according to which one can classify complex networks into categories:^21^ assortative networks, disassortative networks, and uncorrelated networks. For example, social networks are usually assortative, while technological and biological networks disassortative. Furthermore, degree correlations significantly influence the collective dynamical behaviors, including intentional attacks on hub nodes,^22,23^ games,^24^ and synchronization,^25^ to name but a few.

In view of the importance of both the inhomogeneous degree distribution and degree correlations, some fundamental questions rise naturally: In heterogenous correlated networks, how does the structural heterogeneity, characterized by the alterable degree exponent γ, affect the scaling character of the APL d(N)? Does the relation between d(N) and γ in uncorrelated networks also hold for networks with degree correlations? Is the behavior of random walks related to structural heterogeneity in correlated networks? Such a series of important questions still remain open.

In this paper, we study the APL of and a random walk on a family of deterministic treelike disassortative SFNs with changeable degree exponent γ∊(2,1+ln 3/ln 2]. We choose deterministic networks as investigation object because they allow us to study analytically their topological properties and some dynamic processes running on them. Our exact results show that in contrast to the scaling obtained for uncorrelated networks, in their full range of γ, the APL of the considered deterministic networks grows logarithmically with the number of nodes, and that only partial scalings of the random walk depend on the degree exponent γ.

## II. THE RECURSIVE SCALE-FREE TREES

In this section, we introduce a network model defined in a recursive way,^26^ which has attracted a great amount of attention.^27–31^ We call this model *recursive scale-free trees* (RSFTs).^26^ We investigate RSFTs because of their intrinsic interest and because these networks have general degree distribution exponent γ∊(2,1+ln 3/ln 2]. Moreover, RSFTs are deterministic, which allows us to study analytically their topological properties and dynamical processes on them. They are therefore good test beds and substrate networks.

**FIG. 1.**
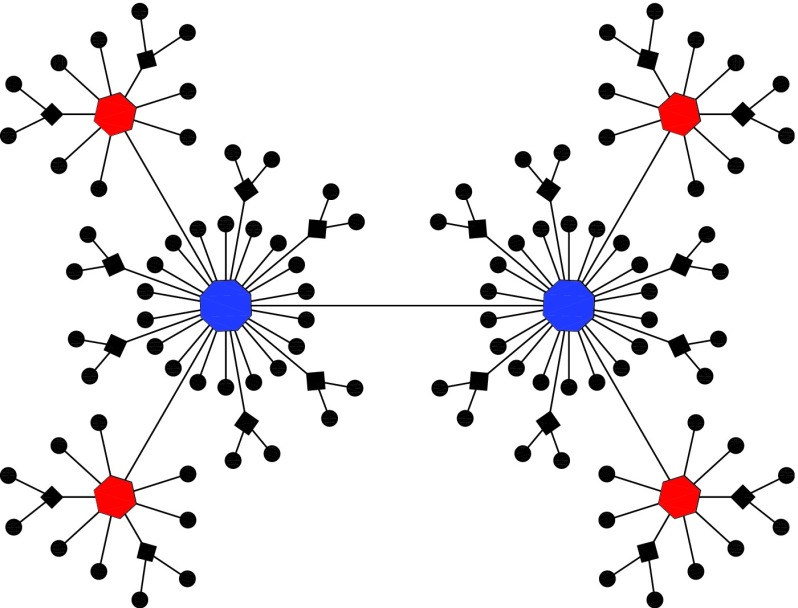
The first four generations of the network construction for a special case of m=2.

The RSFTs, denoted by $R_{m,t}$ (m is a positive integer) after t (t≥0) generation evolution, are constructed as follows. For t=0, $R_{m,0}$ is an edge connecting two nodes. For t≥1, $R_{m,t}$ is obtained from $R_{m,t-1}$: For each of the existing edges in $R_{m,t-1}$, m new nodes are introduced and connected to either end of the edge. Figure 1 shows the construction process for the particular case of m=2.

According to the network construction, one can see that at each step $t_{i}$ $\left(t_{i}\geq 1\right)$, the number of newly introduced nodes is $L_{v}\left(t_{i}\right)=2m{\left(1+2m\right)}^{t_{i}-1}$. From this result, we can easily compute network order (i.e., the total number of nodes) $N_{t}$ at step t,

$$N_{t}=\sum_{t_{i}=0}^{t}L_{v}\left(t_{i}\right)={\left(2m+1\right)}^{t}+1.$$ (1)

Let $k_{i}\left(t\right)$ be the degree of a node i at time t, which entered the networks at step $t_{i}$ $\left(t_{i}\geq 0\right)$. Then

$$k_{i}\left(t\right)={\left(m+1\right)}^{t-t_{i}}.$$ (2)

From Eq. (2), one can easily see that at each step the degree of a node increases m times, i.e.,

$$k_{i}\left(t\right)=\left(m+1\right)k_{i}\left(t-1\right).$$ (3)

RSFTs present some typical characteristics of real-life networks in nature and society, and their main topological properties are controlled by the parameter m. They have a power-law degree distribution with exponent γ=1+ln(2m+1)/ln(m+1) belonging to the interval between 2 and 3.^26,30^ The diameter of RSFTs, defined as the longest shortest distance between any pair of nodes, increases logarithmically with network order,^30^ that is to say, RSFTs are small world. The betweenness distribution exhibits a power-law behavior with exponent $\gamma_{b}=2$.^27^ In addition, RSFTs are disassortative, the average degree of nearest neighbors $k_{\text{nn}}\left(k\right)$ for nodes with degree k is approximately a power-law function of k with a negative exponent.^30^

After introducing the RSFTs, in what follows we will study the average path of the RSFTs and random walks on them. We will show that the exponent γ of degree distribution has no qualitative effect on APL and mean first-passage time (FPT) for all nodes, but has essential influence on FPT for old nodes when the networks grow.

## III. APL

We now study analytically the APL $d_{t}$ of the RSFTs $R_{m,t}$ by using a method similar to but different from that proposed in Ref. 32. It follows that

$$d_{t}=\frac{\Phi_{t}}{N_{t}\left(N_{t}-1\right)/2},$$ (4)

where $\Phi_{t}$ is the total distance between all couples of nodes, i.e.,

$$\Phi_{t}=\sum_{i∊R_{m,t},j∊R_{m,t},i\neq j}d_{ij},$$ (5)

in which $d_{ij}$ is the shortest distance between nodes i and j.

**FIG. 2.**
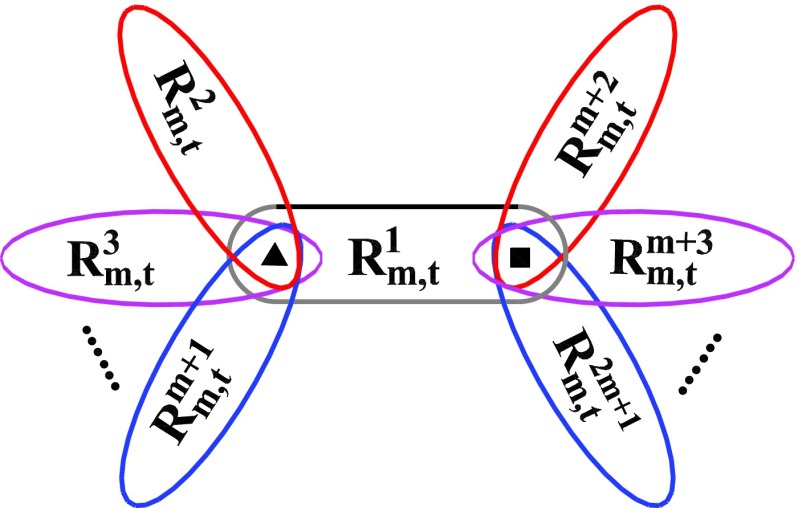
Second construction method of RSFTs. The graphs after t+1 construction steps, $R_{m,t+1}$, may be obtained by the juxtaposition of 2m+1 copies of $R_{m,t}$, denoted by $R_{m,t}^{\theta }$ (θ=1,2,3,⋯,2m,2m+1), which are connected to one another at the border nodes X (▲) and Y (◼).

Notice that in addition to the recursive construction, RSFTs can be alternatively created in another method. Given a generation t, $R_{m,t+1}$ may be obtained by joining at hub nodes 2m+1 copies of $R_{m,t}$, see Fig. 2. The second construction method highlights the self-similarity of RSFTs, which allows us to address $d_{t}$ analytically. From the obvious self-similar structure, it is easy to see that the total distance $\Phi_{t+1}$ satisfies the recursion relation

$$\Phi_{t+1}=\left(2m+1\right)\Phi_{t}+\Delta_{t},$$ (6)

where $\Delta_{t}$ is the sum over all shortest paths whose end points are not in the same $R_{m,t}^{\theta }$ branch. The solution of Eq. (6) is

$$\Phi_{t}={\left(2m+1\right)}^{t-1}\Phi_{1}+\sum_{\tau =1}^{t-1}{\left(2m+1\right)}^{t-\tau -1}\Delta_{\tau }.$$ (7)

The paths contributing to $\Delta_{t}$ must all go through at least either of the two border nodes (i.e., X and Y in Fig. 2) where the different $R_{m,t}^{\theta }$ branches are connected. The analytical expression for $\Delta_{t}$, called the crossing paths, is found below.

Let $\Delta_{t}^{\alpha ,\beta }$ be the sum of all shortest paths with end points in $R_{m,t}^{\alpha }$ and $R_{m,t}^{\beta }$. According to whether or not two branches are adjacent, we split the crossing paths $\Delta_{t}^{\alpha ,\beta }$ into two classes: If $R_{m,t}^{\alpha }$ and $R_{m,t}^{\beta }$ meet at a border node, $\Delta_{t}^{\alpha ,\beta }$ rules out the paths where either end point is that shared border node. For example, each path contributing to $\Delta_{t}^{1,2}$ should not end at node X. If $R_{m,t}^{\alpha }$ and $R_{m,t}^{\beta }$ do not meet, $\Delta_{t}^{\alpha ,\beta }$ excludes the paths where either end point is X or Y. For instance, each path contributive to $\Delta_{t}^{2,m+2}$ should not end at nodes X or Y. We can easily compute that the numbers of the two types of crossing paths are $m^{2}+m$ and $m^{2}$, respectively. On the other hand, any two crossing paths belonging to the same class have identical length. Thus, the total sum $\Delta_{t}$ is given by

$$\Delta_{t}=\left(m^{2}+m\right)\Delta_{t}^{1,2}+m^{2}\Delta_{t}^{2,m+2}.$$ (8)

In order to determine $\Delta_{t}^{1,2}$ and $\Delta_{t}^{2,m+2}$, we define

$$\sigma_{t}=\sum_{i∊R_{m,t},i\neq X}d_{iX}.$$ (9)

Considering the self-similar network structure, we can easily know that at time t+1, the quantity $\sigma_{t+1}$ evolves recursively as

$$\sigma_{t+1}=\left(m+1\right)\sigma_{t}+m\left[\sigma_{t}+\left(N_{t}-1\right)\right]=\left(2m+1\right)\sigma_{t}+m{\left(2m+1\right)}^{t}.$$ (10)

Using $\sigma_{0}=1$, we have

$$\sigma_{t}=\left(mt+2m+1\right){\left(2m+1\right)}^{t-1}.$$ (11)

Having obtained $\sigma_{t}$, the next step is to compute the quantities $\Delta_{t}^{1,2}$ and $\Delta_{t}^{2,m+2}$ given by

$$\Delta_{t}^{1,2}=\sum_{\begin{array}{c} i∊R_{m,t}^{1},j∊R_{m,t}^{2}\\ i,j\neq X \end{array}}d_{ij}=\sum_{\begin{array}{c} i∊R_{m,t}^{1},j∊R_{m,t}^{2}\\ i,j\neq X \end{array}}\left(d_{iX}+d_{jX}\right)=\left(N_{t}-1\right)\sum_{\begin{array}{c} i∊R_{m,t}^{1}\\ i\neq X \end{array}}d_{iX}+\left(N_{t}-1\right)\sum_{\begin{array}{c} j∊R_{m,t}^{2}\\ j\neq X \end{array}}d_{jX}=2\left(N_{t}-1\right)\sigma_{t}$$ (12)

and

$$\Delta_{t}^{2,m+2}=\sum_{\begin{array}{c} i∊R_{m,t}^{2},i\neq X\\ j∊R_{m,t}^{m+2},j\neq Y \end{array}}d_{ij}=\sum_{\begin{array}{c} i∊R_{m,t}^{2},i\neq X\\ j∊R_{m,t}^{m+2},j\neq Y \end{array}}\left(d_{iX}+d_{XY}+d_{jY}\right)=2\left(N_{t}-1\right)\sigma_{t}+{\left(N_{t}-1\right)}^{2},$$ (13)

where $d_{XY}=1$ has been used. Substituting Eqs. (12) and (13) into Eq. (8), we obtain

$$\Delta_{t}=2m\left(2m+1\right)\left(N_{t}-1\right)\sigma_{t}+m^{2}{\left(N_{t}-1\right)}^{2}=m\left(2mt+5m+2\right){\left(2m+1\right)}^{2t}.$$ (14)

Inserting Eqs. (14) for $\Delta_{\tau }$ into Eq. (7), and using $\Phi_{1}=5m^{2}+4m+1$, we have

$$\Phi_{t}=\frac{{\left(2m+1\right)}^{t-1}}{2}\left[1+m+\left(2mt+3m+1\right){\left(2m+1\right)}^{t}\right].$$ (15)

Plugging Eq. (15) into Eq. (4), one can obtain the analytical expression for $d_{t}$,

$$d_{t}=\frac{1+m+\left(2mt+3m+1\right){\left(2m+1\right)}^{t}}{\left(2m+1\right)\left[{\left(2m+1\right)}^{t}+1\right]},$$ (16)

which approximates 2mt/(2m+1) in the infinite t, implying that the APL shows a logarithmic scaling with network order. Therefore, RSFTs exhibit a small-world behavior. Notice that this scaling has been seen previously in some other deterministic disassortative SFNs in the same exponent range, such as the pseudofractal scale-free web studied in Refs. 33 and 34 and the “transfractal” recursive networks addressed in Ref. 35.

**FIG. 3.**
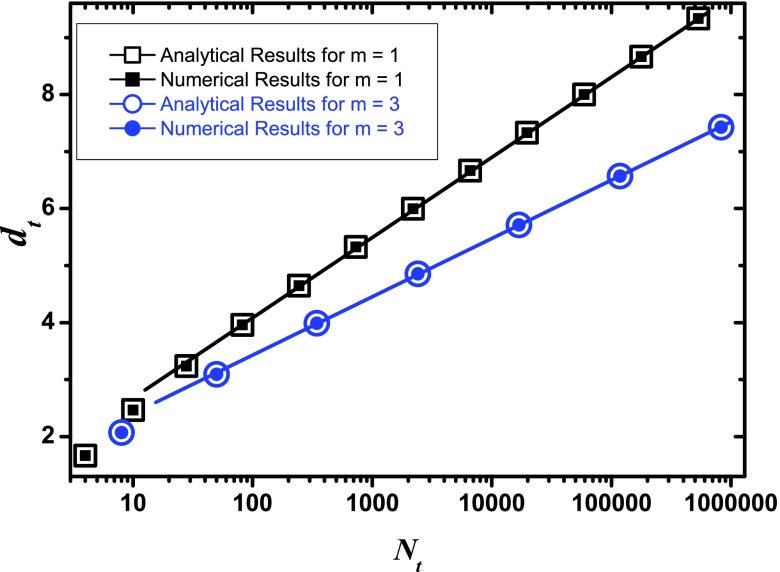
APL $d_{t}$ vs network order $N_{t}$ on a semilog scale. The solid lines are guides to the eyes.

We have checked our analytic result against numerical calculations for different m and various t. In all the cases we obtain a complete agreement between our theoretical formula and the results of numerical investigation, see Fig. 3.

The logarithmic scaling of APL with network order in full rage of degree exponent γ shows that previous relation between APL and γ obtained for uncorrelated SFNs^7,8^ is not valid for disassortative SFNs, at least for RSFTs and some other deterministic scale-free graphs. This leads us to the conclusion that degree exponent γ itself does not suffice to characterize the APL of SFNs.

## IV. RANDOM WALKS

This section considers simple random walks on RSFTs defined by a walker such that at each step the walker, located on a given node, moves to any of its nearest neighbors with equal probability.

### A. Scaling efficiency

We follow the concept of *scaling efficiency* introduced in Ref. 11. Denote by $T_{ij}$ the mean FPT between two nodes i and j. Let $T_{ii}$ be the mean time for a walker returning to a node i for the first time after the walker have left it. When the network order grows from N to gN, one expects that in the infinite limit of N,

$$T_{ij}\left(gN\right)∼g^{\delta_{ij}}T_{ij}\left(N\right),$$ (17)

where $\delta_{ij}$ is defined as the scaling efficiency exponent. An analogous relation for $T_{ii}$ defines exponent $\delta_{ii}$.

One can confine scaling efficiency in the nodes already existing in the networks before growth. Let $T_{ij}^{\prime }\left(gN\right)$ be the mean FPT in the networks under consideration, averaged over the *original* class of nodes (before growth). Then the *restricted* scaling efficiency exponent $\lambda_{ij}$ is defined by relation

$$T_{ij}^{\prime }\left(gN\right)∼g^{\lambda_{ij}}T_{ij}\left(N\right).$$ (18)

Similarly, we can define $\lambda_{ii}$.

After introducing the concepts, in the following we will investigate random walks on RSFTs following a similar method used in Ref. 11. It should be mentioned that our motivation is different from that of Ref. 11. In the work of Ref. 11 the authors analyzed SFNs with a single degree distribution exponent γ=1+ln 3/ln 2, the purpose of that work is to find what is special about random walks on SFNs, compared to other types of graphs. Here we study random walks on SFNs (RSFTs) with general γ∊(2,1+ln 3/ln 2]. Our aim is to study the effect of degree exponent γ on random walks characterized by the scaling efficiency proposed in Ref. 11.

### B. FPT for old nodes

Consider an arbitrary node i in the RSFTs $R_{m,t}$ after t generation evolution. From Eq. (2), we know that upon growth of RSFTs to generation t+1, the degree $k_{i}$ of node i increases m times, namely, from $k_{i}$ to $\left(m+1\right)k_{i}$. Let the FPT for going from node i to any of the $k_{i}$ old neighbors be T. Let the FPT for going from any of the $mk_{i}$ new neighbors to one of the $k_{i}$ old neighbors be A. Then we can establish the following equations:

(19) $$T=\frac{1}{m+1}+\frac{m}{m+1}\left(1+A\right),$$

A=1+T,

which leads to T=2m+1. Therefore, the passage time from any node i $\left(i∊R_{m,t}\right)$ to any node j $\left(j∊R_{m,t}\right)$ increases 2m times, on average, upon growth of the networks to generation t+1, i.e.,

$$T_{ij}^{\prime }\left(N_{t+1}\right)=\left(2m+1\right)T_{ij}\left(N_{t}\right).$$ (20)

Since the network order approximatively grows by 2m times in the large t limit, see Eq. (1). This indicates that the scaling efficiency exponent for old nodes is $\lambda_{ij}=1$, which is a constant independent of the degree exponent γ.

Next we continue to consider the return FPT to node i. Denote by $T_{ii}^{\prime }$ the FPT for returning to node i in $R_{m,t+1}$. Denote by $T_{ji}^{\prime }$ the FPT from j—an old neighbor of i $\left(i∊R_{m,t}\right)$—to i, in $R_{m,t+1}$. Analogously, denote by $T_{ii}$ the FPT for returning to i in $R_{m,t}$, and $T_{ji}$ the FPT from the same neighbor j, to i, in $R_{m,t}$. For $R_{m,t}$, we have

$$T_{ii}=\frac{1}{k_{i}}\sum_{j∊\Omega_{i}\left(t\right)}\left(1+T_{ji}\right)=1+\frac{1}{k_{i}}\sum_{j∊\Omega_{i}\left(t\right)}T_{ji},$$ (21)

where $\Omega_{i}\left(t\right)$ is the set of neighbors of node i, which belong to $R_{m,t}$. On the other hand, For $R_{m,t+1}$,

$$T_{ii}^{\prime }=\frac{m}{m+1}\times 2+\frac{1}{m+1}\frac{1}{k_{i}}\sum_{j∊\Omega_{i}\left(t\right)}\left(1+T_{ji}^{\prime }\right).$$ (22)

The first term on the right-hand side of Eq. (22) accounts for the process in which the walker moves from node i to the new neighbors and back. Since among all neighbors of node i, m/(m+1) of them are new, which is obvious from Eq. (3), such a process occurs with a probability m/(m+1) and takes two time steps. The second term on the right-hand side interprets the process where the walker steps from i to one of the old neighbors previously existing in $R_{m,t}$ and back, this process happens with the complimentary probability 1−m/(m+1)=1/(m+1).

Using Eq. (20) to simplify Eq. (22), we can obtain

$$T_{ii}^{\prime }=\frac{2m+1}{m+1}T_{ii}={\left(2m+1\right)}^{\left[1-\text{ln}\left(m+1\right)/\text{ln}\left(2m+1\right)\right]}T_{ii}.$$ (23)

In other words,

$$T_{ii}^{\prime }\left(N_{t+1}\right)={\left(2m+1\right)}^{\lambda_{ii}}T_{ii}\left(N_{t}\right),$$ (24)

where the scaling efficiency exponent $\lambda_{ii}=1-\text{ln}\left(m+1\right)/\text{ln}\left(2m+1\right)=1-1/\left(\gamma -1\right)$ is an increasing function of γ. Thus, the more heterogeneous the network structure, the more easily for the walker to return to the origin when the networks grow in size.

### C. FPT for all nodes

We now compute $T_{j^{\prime }j^{\prime }}$, the FPT to return to a new node $j^{\prime }∊R_{m,t}$, that is, a neighbor of node $i∊R_{m,t-1}$. Denote by $T_{1}$ the FPT from i to $j^{\prime }$, and B the FPT to return to i (starting off from i) without ever visiting $j^{\prime }$. Then we have

$$T_{j^{\prime }j^{\prime }}=1+T_{1}$$ (25)

and

$$T_{1}=\frac{1}{k_{i}}+\frac{k_{i}-1}{k_{i}}\left(B+T_{1}\right).$$ (26)

Equation (26) can be interpreted as follows: With probability $1/k_{i}$ ($k_{i}$ being the degree of node i in $R_{m,t}$), the walker starting from node i would take one time step to go to node $j^{\prime }$; with the complimentary probability $\left(k_{i}-1\right)/k_{i}$, the walker chooses uniformly a neighbor node except $j^{\prime }$ and spends on average time B in returning to i, then takes time $T_{1}$ to arrives at node $j^{\prime }$.

In order to close Eqs. (25) and (26), we express the FPT to return to i as

$$T_{ii}\left(N_{t}\right)=\frac{1}{k_{i}}\times 2+\frac{k_{i}-1}{k_{i}}B.$$ (27)

Eliminating $T_{1}$ and R, we obtain

$$T_{j^{\prime }j^{\prime }}\left(N_{t}\right)=k_{i}T_{ii}\left(N_{t}\right).$$ (28)

Combining Eqs. (3), (23), and (28), we have

$$T_{j^{\prime }j^{\prime }}\left(N_{t}\right)=2{\left(2m+1\right)}^{t}∼2N_{t}.$$ (29)

Iterating Eqs. (23) and (28), we have that in $R_{m,t}$ there are $L_{v}\left(\epsilon \right)$ (0≤ϵ≤t) nodes with $T_{ii}=2{\left(2m+1\right)}^{t}/{\left(m+1\right)}^{t-\epsilon }$. Taking average of $T_{ii}$ over all nodes in $R_{m,t}$ leads to

$${\left\langle T_{ii}\right\rangle }_{t}=\frac{1}{{\left(2m+1\right)}^{t}+1}\left[\frac{4m{\left(2m+1\right)}^{t}}{{\left(m+1\right)}^{t}}+\frac{4m{\left(2m+1\right)}^{t}}{{\left(m+1\right)}^{t-1}}\times \frac{{\left(2m+1\right)}^{t}{\left(m+1\right)}^{t}-1}{m\left(2m+3\right)}\right]\vec{t\to \infty }\frac{4m+4}{2m+3}{\left(2m+1\right)}^{t}∼\frac{4m+4}{2m+3}N_{t}.$$ (30)

Equation (30) implies that $\delta_{ii}=1$, which is uncorrelated with the degree exponent γ.

We continue to calculate $T_{ij}$ in $R_{m,t}$, which is FPT from an arbitrary node i to another node j. Since each of the newly created nodes has a degree of 1 and is linked to an old node, the FPT $T_{i^{\prime }j}$ from node $i^{\prime }$—a new neighbor of the old node i—to j equals $T_{ij}+1$ and thus has little effect on the scaling when network order N is very large. Therefore, we need only to consider FPT $T_{ij^{\prime }}$ from i to $j^{\prime }$—a new neighbor of j, which can be expressed as

$$T_{ij^{\prime }}=T_{ij}+T_{jj^{\prime }}.$$ (31)

Notice that

$$T_{j^{\prime }j^{\prime }}=1+T_{jj^{\prime }}.$$ (32)

Substituting Eqs. (32) and (29) for $T_{j^{\prime }j^{\prime }}$ into Eq. (31) results in

$$T_{ij^{\prime }}=T_{ij}+2{\left(2m+1\right)}^{t}-1∼N_{t},$$ (33)

where Eq. (20) has been used. Therefore, we have

$${\left\langle T_{ij}\right\rangle }_{t}∼N_{t},$$ (34)

which shows that mean transit time between arbitrary pair of nodes is proportional to network order. Equation (34) also reveals that $\delta_{ij}$ is a constant 1, which does not depend on γ.

## V. CONCLUSIONS

To explore the effect of structural heterogeneity on the scalings of APL and random walks occurring on disassortative SFNs, we have studied analytically a class deterministic SFNs—RSFTs—with various degree exponents γ. In addition to scale-free distribution, RSFTs also reproduce some other remarkable properties of many natural and man-made networks: small APL, power-law distribution of betweenness distribution, and negative degree correlations. They can thus mimic some real systems to some extent.

With the help of recursion relations derived from the self-similar structure, we have obtained the solution of APL for RSFTs. In contrast to the well-known result that for uncorrelated SFNs with network order N and degree exponent 2≤γ<3, their APL d(N) behaves as a double logarithmic scaling, d(N)∼ln ln N, our rigorous solution shows that the APL of RSFTs behaves as a logarithmic scaling, in despite of their degree exponent γ∊(2,1+ln 3/ln 2]. Therefore, degree correlations have a profound impact on the APL of SFNs.

We have also investigated analytically random walks on RSFTs. We have shown that for the full range of γ, the mean transit time $T_{ij}\left(N\right)$ between two nodes averaged over all node pairs grows linearly with network order N. The same scaling holds for the FPT $T_{ii}\left(N\right)$ for returning back to the origin i after the walker has started from i. Thus, despite different γ, all the RSFTs exhibit identical scalings of FPT and return FPT for all nodes. On the other hand, for those nodes already existing in the networks before growth, the restricted scaling efficiency exponents are $\lambda_{ij}=1$ and $\lambda_{ii}=1-1/\left(\gamma -1\right)$, where $\lambda_{ij}$ is not pertinent to γ, but $\lambda_{ii}$ depends on γ.

We should stress that our conclusions were drawn only from a particular type of deterministic treelike disassortative networks. It is still unknown whether the conclusions are also valid for stochastic disassortative networks, especially for networks in the presence of loops. But our results may provide some insights into random walk problem on complex networks, in particular, on trees. More recently, the so-called border tree motifs have been shown to be significantly present in real networks,^36^ looking from this angle, our work may also shed light on some real-world systems. Finally, we believe that our analytical techniques could be helpful for computing APL of and transit time for random walks on other deterministic media. Moreover, since exact solutions can serve for a guide to and a test of approximate solutions or numerical simulations, we also believe that our vigorous closed-form solutions can prompt related studies of random networks.
